# How do geriatricians feel about managing older people living with HIV? A scoping review

**DOI:** 10.1007/s41999-022-00642-4

**Published:** 2022-04-09

**Authors:** Howell T. Jones, Tristan J. Barber

**Affiliations:** 1 Ian Charleson Day Centre, Royal Free London NHS Foundation Trust, Pond Street, Hampstead, London, NW3 2QG UK; 2 MRC Unit for Lifelong Health and Ageing at UCL, London, UK; 3 Institute for Global Health, UCL, London, UK

**Keywords:** Frailty, HIV, AIDS, Geriatrics, Older people

## Abstract

**Purpose:**

The proportion of people living with HIV being older adults is increasing and due to high rates of multimorbidity and frailty within this group geriatricians are well placed to contribute to their care. However, little is known about how geriatricians feel about this new opportunity.

**Methods:**

A scoping review was performed following the Arksey & O’Malley’s methodological framework with nine databases searched in December 2021 for studies reporting the experiences or views of geriatricians on caring for people living with HIV. Study inclusion was not limited by language or year of publication. Narrative reviews were excluded. Two reviewers independently performed the extraction using predetermined criteria. A descriptive analysis of extracted information was performed.

**Results:**

Six publications reporting four studies, all conducted in the USA, were identified. The current barriers to geriatricians being involved in the care of older people living with HIV are: their current experience in managing people living with HIV, their knowledge of HIV, specific issues related to older people living with HIV and screening for HIV in older people as well as their attitudes to people living with HIV and experience of managing older LGBTQ + people.

**Conclusion:**

Prior to geriatricians being routinely involved in the care of older people living with HIV further research outside of the USA is required. Geriatricians will also require specific training which should be incorporated into geriatric medicine training curricula as well as the creation of learning tools and quality clinical practice guidelines ideally created in collaboration with HIV organisations.

**Supplementary Information:**

The online version contains supplementary material available at 10.1007/s41999-022-00642-4.

## Key summary points

**Aim:**

To identify what evidence exists regarding how geriatricians feel about managing older people living with HIV.

**Findings:**

Currently whilst geriatricians are willing to contribute to the care of older people living with HIV, they lack the experience and training to take a prominent role.

**Message:**

Incorporating education about HIV care into geriatric medicine curricula and the formation of quality clinical practice guidelines is required to prepare geriatricians to help participate in the care of older people living with HIV.

**Supplementary Information:**

The online version contains supplementary material available at 10.1007/s41999-022-00642-4.

## Introduction

Across Europe approximately half of the people accessing HIV services are over the age of 50 and by 2030 this will rise to 70% with almost 40% being over 65 [1–5]. The reasons for this rise include the availability of effective antiretroviral therapy (ART) and improved management of opportunistic infections and comorbidities [6, 7]. However, another factor is that the number of new diagnoses in older people is increasing, with one in six new cases of HIV diagnosed in Europe being in someone aged 50 or older, representing 14% of new diagnoses with this increasing by 2% annually [1, 8].

HIV is associated with high rates of multimorbidity and frailty making geriatricians well positioned to contribute to the care of people living with HIV which is presently done within HIV services by clinicians less familiar with the concept of ‘Comprehensive Geriatric Assessment’ (CGA) as evidenced by of 23 out of 98 HIV services in the United Kingdom (UK) surveyed in 2016 reporting a need for a dedicated ageing service and approximately half of clinics indicating they would refer complex older adults to a geriatrician [9–14]. The most recent European guidelines produced by the European AIDS Clinical Society (EACS) emphasises the importance of frailty screening and CGA whilst those produced by the British HIV Association (BHIVA) promote incorporating geriatricians into the care of complex older people living with HIV [15, 16].

Currently in some European countries geriatricians are already involved in the delivery of specialist clinics for older people living with HIV and report positive outcomes suggesting a multidisciplinary model could prevent older people living with HIV falling between the cracks in existing services [17, 18]. However, this will require more geriatricians to become familiar with HIV and how it affects people in later life. Whilst numerous papers highlight the importance of CGA and the engagement of geriatricians in the care of older people living with HIV little is known about how geriatricians themselves feel about this new opportunity [3, 6, 14, 19–22].

## Methods

A scoping review was performed according to the methodological framework developed by Arksey and O’Malley with reporting following the Preferred Reporting Items for Systematic Reviews and Meta-Analyses (PRISMA) Extension for Scoping Review checklist [23, 24].

### Search strategy

A comprehensive search of published research was conducted in December 2021 with nine computerised databases (AMED, BNI, CINAHL, EMBASE, EMCARE, HMIC, Medline, PsychINFO and PubMed) accessed using synonyms of the keywords ‘HIV’ and ‘geriatrician’ or ‘geriatric medicine’. The grey literature was also searched. The full search strategy is outlined in Appendix 1.

### Identifying the research question

The research question was ‘what evidence exists regarding how geriatricians feel about managing older people living with HIV?’.

### Study selection

The search was performed using the Healthcare Databases Advanced Search (HDAS) and after duplicates were removed the two reviewers (HTJ and TJB) independently assessed the titles and abstracts for eligibility. Further review of potentially eligible full texts was then done against the eligibility criteria by both reviewers. There was a strategy in place to engage a third reviewer should differences in opinion during the selection process have arisen.

### Eligibility criteria

#### Population

Any studies exploring the views of doctors working in the field of geriatric medicine on managing people living with HIV were included regardless of their stage of training. No other limitations based on population characteristics were applied.

#### Concept

Any publications reporting the desired outcome measure were included regardless of their primary aims.

#### Context

All sources of evidence pertaining to any country or contextual setting were eligible for inclusion.

### Type of evidence sources

The initial search was limited to primary research articles or systematic reviews from peer reviewed sources as despite scoping reviews not requiring appraisal of methodological quality, the peer review process ensures the research question is answered through robust data. Due to the limited number of relevant articles, a decision was made to include peer reviewed conference abstracts and search the grey literature for additional studies with the intent to consolidate all existing data. Narrative reviews were excluded as they do not report original results but their reference lists were reviewed to identify additional eligible studies. There was no limitation on the year or language of publication to allow worldwide studies to be included as well as information from both the pre-ART and post-ART eras.

### Data charting

The reviewers determined what data was to be extracted prior to data charting to maintain consistency. Extracted data included: title, authors, publication type, journal, publication year, location and country of study, study aims, sample size and demographics, study design, analysis methodology as well as key findings. Extracted data were examined by both reviewers for clarity and reliability.

### Quality appraisal

As this was a scoping review which aims to identify gaps in existing evidence, methodological quality was not assessed.

### Data analysis

Extracted information was tabulated according to the categories outlined above, with a descriptive analysis of extracted information performed and presented narratively.

## Results

### Selection of studies

**Fig. 1:**
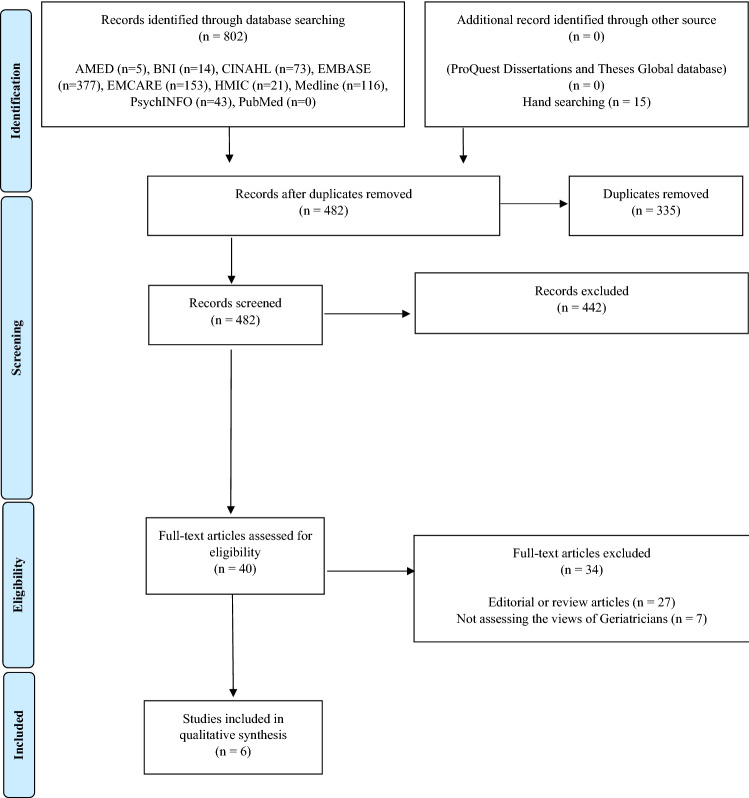
PRISMA Diagram. *From:* Moher D, Liberati A, Tetzlaff J, Altman DG, The PRISMA Group (2009). *P*referred *R*eporting *I*tems for *S*ystematic Reviews and *M*eta-*A*nalyses: The PRISMA Statement. PLoS Med 6(7): e1000097. https://doi.org/10.1371/journal.pmed1000097. For more information, visit www.prisma-statement.org

The initial database search yielded 802 results which was reduced to 467 after duplicate removal, with an additional 15 articles identified through hand searching. Screening records by title and abstract resulted in 40 full-text articles being retrieved with six proving eligible for analysis. No further studies were identified from the reference lists of included studies or from the grey literature. The full PRISMA flow diagram is displayed in Fig. 1.

### Study characteristics

The identified studies were published between October 2008 and April 2021 with three (50%) being published in the last 5 years (Table 1) [25–30]. All studies were conducted within the Unites States of America (USA) [25–30]. Five publications (83%) were conference abstracts with the remaining being a primary research article [25–30]. Three publications presented the results of the same study (two conference abstracts and one article) [27, 28, 30]. Five (83%) utilised a cross-sectional methodology involving surveys, whilst the remaining study was a pilot of an elective within HIV medicine for geriatric medicine trainees [25–30]. The participants of the studies were broad with three (the aforementioned same study) involving geriatricians, as well as nurses and social workers [27, 28, 30]. One study included staff at both a family medicine and geriatric medicine practice whilst the last presented the views of geriatric medicine providers only [26, 29]. Most studies did not report full participant demographic data but in those that did geriatricians were predominately White (62–66%) females (57–64%) who were heterosexual (93.7%) and who had been working in geriatric medicine for an average of 10 years [27, 28].

**Table 1:** Summary of included articles

ID	Title	Year	Location	Aim	Sample	Method
1	Creation and pilot implementation of a geriatrics fellows’ elective in HIV medicine for older adults [25]	2020	Chicago, IL, USA	To evaluate a pilot curriculum for geriatric medicine trainees in HIV Medicine	1 geriatric medicine fellow	Feedback on elective
2	Enhancing cultural and medical competency for LGBT older adults [26]	2021	Philadelphia, PA, USA	To assess assessed self-reported knowledge and attitudes related to culturally competent care for LGBT patients and respondents comfort providing medical care for LGBT patients	57 providers from one family medicine and one geriatric medicine practice	A voluntary, anonymous on-line survey was emailed to providers and staff at one urban, family medicine and one geriatric practice
3	Geriatricians and HIV: Comfort level and knowledge [27]	2008	Baltimore, MD, USA	To assess geriatricians' knowledge of and comfort with HIV/AIDS	94 geriatricians	A self-administered survey was mailed to a random sample of 302 US physicians who indicated that their primary area of practice was geriatrics
4	HIV knowledge and attitudes among providers in aging: results from a national survey [28]	2011	East Lansing, MI, USA	To examine knowledge of HIV/AIDS and attitudes toward people with HIV/AIDS in a national sample of physicians, nurses, and social workers who specialize in gerontology or geriatrics	111 geriatricians (Total *n* = 486)	Cross-sectional survey of members of the American Medical Association (AMA), the National Association of Social Workers (NASW), and the American Nursing Credentialing Center (ANCC) The survey instrument included three standardized measures: 1. HIV Knowledge Questionnaire-45 (HIV-K-Q-45) 2. AIDS Attitude Scale (AAS) 3. A short version of the Marlowe-Crowne Social Desirability Scale (MCSDS) Additional questions specifically on older adults: 1. Whether or not they thought dementia due to AIDS was reversible with appropriate treatment 2. Whether or not the proportion of older adults contracting HIV through heterosexual transmission was decreasing 3. What percent of all AIDS cases in the USA have occurred in people aged 50 and over 4. To correctly rank the four most common risk factors for HIV
5	HIV screening in an urban geriatrics ambulatory clinic [29]	2020	New York City, NY, USA	To measure the rate of HIV screening among older patients as well as assess provider knowledge for HIV testing guidelines in an urban safety netgeriatrics clinic	14 geriatricians	Brief survey of geriatricians
6	Learning what we don’t know: Attitudes and knowledge of gerontological health providers toward HIV/AIDS [30]	2010	East Lansing, MI, USA	To assess provider knowledge and attitudes toward HIV/AIDS	111 geriatricians (Total *n* = 486)	A survey of knowledge and attitudes was mailed to providers of gerontological health services across the USA

### Findings

**Fig. 2:**
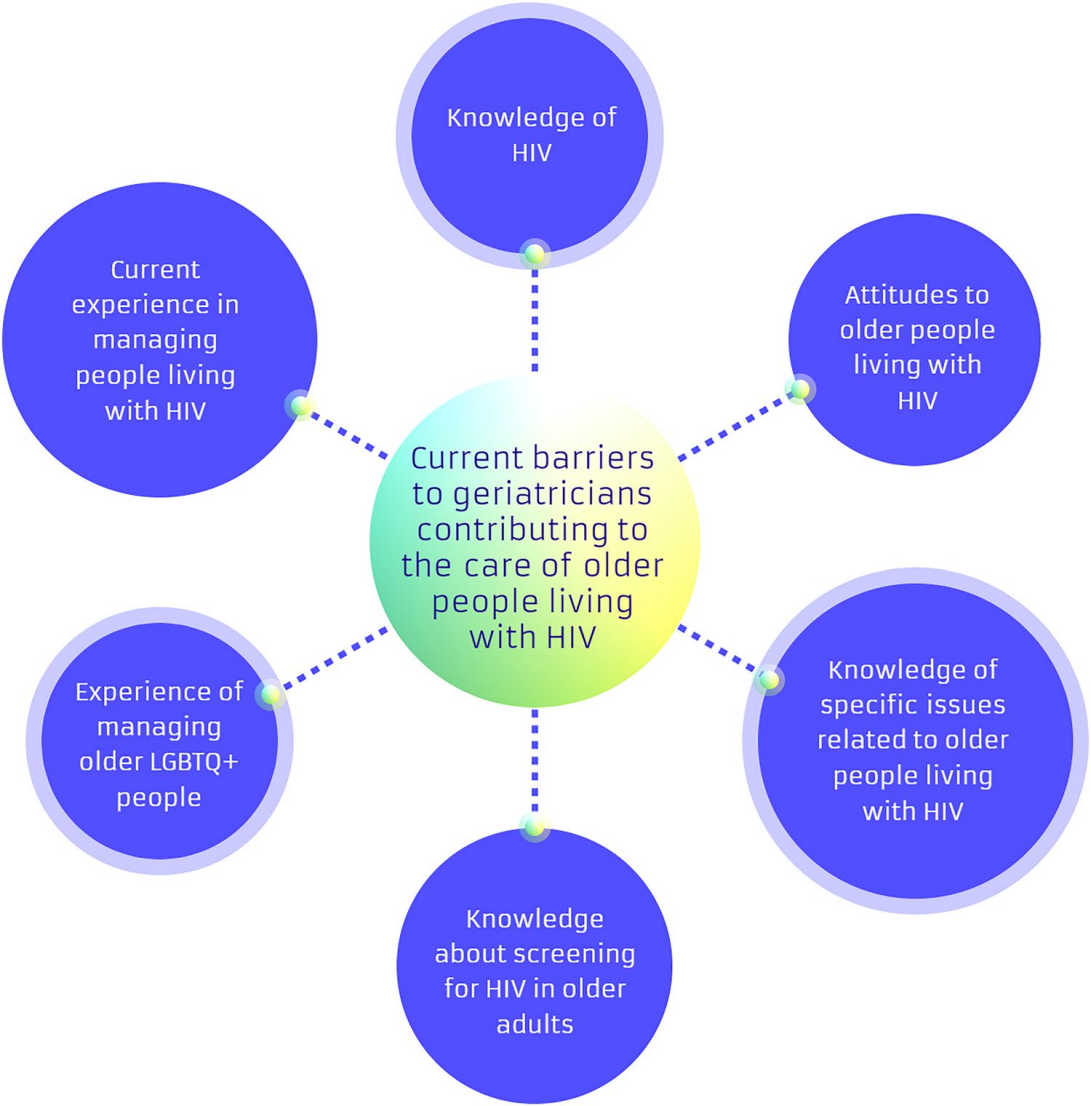
Current barriers to geriatricians contributing to the care of older people living with HIV

Six main themes were identified from the literature (Fig. 2):

#### Current experience in managing people living with HIV

In a cohort of 94 geriatricians 46% reported they were ‘not comfortable at all’ in providing care to people living with HIV, with 44.7% having seen none in the last year and the remaining 46.8% seeing between one and five, which explains the predominant finding from all studies being that currently geriatricians have a knowledge gap related to the management of people living with HIV [25–30].

#### Knowledge of HIV

Hughes utilised the original ‘HIV Knowledge Questionnaire-45’ (HIV-K-Q-45) which is a self-administered 45 part true or false questionnaire developed to assess respondents knowledge of HIV [31]. They compared the results of geriatricians (*n* = 111), to nurses (*n* = 190) and social workers (*n* = 173) and found that geriatricians scored statistically significantly higher (*p* < 0.0005) with a mean score of 39.94 (total sample 38.08) [28, 30].

#### Knowledge of specific issues related to older people living with HIV

Hughes also asked participants what percentage people over 50 contributed to the total number of AIDS cases in the USA from the start of the epidemic to 2006 with scores calculated as the absolute difference from the correct value (13%) with geriatricians scoring 11.2 points from this (total sample: 13.64 points, SD: 12.43, range 0–69) but no statistically significant differences was found between the groups of professionals (*p* = 0.061) [28]. Only 47% of geriatricians were able to correctly rank the four most common risk factors for HIV infection in older people (correct ranking at the time: 1. sex between men, 2. injection drug use 3. sex between men and women 4. blood transfusion) [28, 30]. It must be noted that sex between men and women is now the second most common mode of transmission and injection drug use third [32]. Similarly, only a minority (31%) knew that dementia related to HIV is reversible [27, 28, 30]. Conversely, 75% of geriatricians recognised that cases of HIV amongst heterosexual people over 50 are increasing compared to only 66% of nurses and social workers though again this was not statistically significant (*p* = 0.105) [27, 28, 30]. Finally, a geriatric medicine trainee working with people living with HIV identified that geriatric syndromes were common and presented earlier [25].

#### Knowledge of screening for HIV in older adults

With regards to screening for HIV in a study of 14 geriatric medicine providers 71% reported good knowledge of the United States Preventive Services Task Force (USPSTF) HIV screening guidelines and ordered tests if they identified risk factors [29]. However of the 1259 patients seen in the geriatric medicine clinic in a 3-month period only 31 (2.5%) were tested, with 26 (84%) being tested based on a known risk factor [29]. The mean age of the 31 tested was 81.64, and 14 (45%) of the patients were male with only 7 (22.5%) having documentation of their sexual activity [29]. Respondents reported that annual HIV screening was often not done due to there “not being enough time to discuss” (21%) or because “patients refused to discuss” (21%) [29]. Overall, geriatricians were likely to enquire about older people’s sexual practices less than once a year but when it was done discussion of condom use was common (79%) [29].

#### Attitudes to older people living with HIV

Overall, the geriatricians surveyed had positive attitudes towards older people living with HIV and were attitudinally prepared to provide care to them [26, 28, 30]. Hughes assessed this in their study using a modified version of the ‘AIDS Attitude Scale (AAS)’ which has been shown to be a valid and reliable measure of attitudes, and consists of two subscales: a 14-item avoidance scale and a 7-item empathy scale with each of the 21 items rated on a 6-point Likert scale from 1 (strongly disagree) to 6 (strongly agree) [28, 30, 33, 34]. Mean scores for the AAS subscales range from 1 to 6, with a score of 6 on empathy representing high levels of empathy, while a 6 on the avoidance subscale represents a high level of avoidance [33, 34]. The attitudes of geriatricians were found to be positive with high mean score on the empathy subscale of 5.46 (SD 0.69) and a low mean score on the avoidance subscale of 1.72 (SD 0.69) [28, 30]. However, significant differences were not found for either subscale when comparing the geriatricians to nurses or social workers [28, 30].

#### Experience of managing older LGBTQ + people

Finally, the geriatricians surveyed identified an overlap between people living with HIV and those identifying as lesbian, gay, bisexual, transgender, queer or questioning (LGBTQ +) and had positive attitudes towards caring for them though reported less comfort around trans patients due to concerns about ensuring they used the appropriate pronouns and their lack of experience of gender-affirming hormone therapy [26]. Lastly, of the 57 geriatricians surveyed 39% reported witnessing discrimination towards LGBTQ + patients, families, or staff in the workplace [26].

## Discussion

The recommendations from the six publications were homogenous highlighting that whilst geriatricians have positive attitudes towards people living with HIV and have some understanding of the condition, they currently require further training before they can play a prominent role in the care of older people living with HIV [25–30]. Education should focus on the history of the HIV epidemic and training on HIV as a condition but more specifically how it affects people in older age [25–30].

The richest data came from the 2011 study by Hughes which whilst informative is now over a decade old [28]. Due to ongoing advancements in HIV care, evolving attitudes towards people living with HIV and changes to the demographics of new cases this data is not directly applicable today. The HIV-K-Q-45 is now over 20 years old and therefore contains outdated questions and concepts, for example asking if ‘a person can get HIV from a toilet seat’ [31, 35]. Similarly, the AAS was only validated in groups of nurses, health education students and lay people but never doctors, or specifically geriatricians, meaning the results from Hughes’ study are less reliable as one would expect geriatricians to have a higher level of knowledge of HIV compared to nurses and social workers that could directly impact attitudes [33, 34, 36, 37]. The questions in the AAS are also outdated with several only exploring attitudes towards gay men or people who inject drugs and equating that with being synonymous with living with HIV [33, 34, 36, 37]. These flaws restrict the clinical use of the AAS as they limit its ability to measure one attitude construct at a time, specifically attitudes towards people living with HIV, when the items bring in attitudes about differing constructs such as homosexuality [33, 34, 36, 37]. Attitudes towards LGBTQ + people have progressed since the validation studies for the AAS and Hughes’ study meaning their results have less pertinence today [28, 33, 34, 36, 37]. It must also be noted that Hughes chose to compare knowledge of and attitudes towards people living with HIV between geriatricians, nurses and social workers which are discrete professions [27, 28, 30]. This is important as there are external societal factors that impact the profession a person may pursue. Across all three professions being White was the predominant ethnicity though the geriatrician group had the most diversity with 40.5% being non-White compared to only 11.9% of nurses and 12.9% of social workers [28]. Other differences include more gender variation amongst geriatricians with 57.1% being female versus 96.9% of nurses and 84.2% of social workers and whilst all the nurses recruited identified as heterosexual 6.3% of geriatricians and 7.4% of social workers identified as LGBTQ + [28]. Finally, as expected the geriatrician group had the highest level of postgraduate education, followed by social workers then nurses [28]. Each of these characteristics may impact a person’s knowledge or attitude to a subject matter meaning the groups are not the most optimal comparators. Therefore, it may have been preferable to compare different medical professionals for example geriatricians to infectious diseases or internal medicine specialists. In a 2016 study of infectious disease fellows from across the USA 51% reported little experience in initiating and monitoring patients on ART and only 22% felt this was adequality taught during their fellowship programme [38]. Meanwhile, a 2010 study utilising a cross-sectional survey of 223 s and third year internal medicine residents from four programs in Baltimore, Boston, Detroit, and New York City USA between March and June 2006 identified that 51% had contributed to the inpatient care of at least 30 people living with HIV in the past year whilst the majority (63%) and only cared for up to five outpatients [39]. The majority (89%) of residents viewed managing people living with HIV as an excellent educational opportunity but felt less prepared to do so in an outpatient setting which is important as HIV continues to transition to a chronic illness model [39]. Therefore, the potential hardships geriatricians may face are not specific to this discipline and extend to other non-HIV specialists managing people living with HIV [38, 39]. However, despite its weaknesses Hughes’ work does provide the most robust answer to the research question currently available supporting the need for further studies [28]. Rather than replicating Hughes’ study in Europe what is required is the development of a validated tool to assess clinicians’ knowledge of managing older people living with HIV focussing on current common clinical scenarios such as comorbidities, geriatric syndromes, polypharmacy and drug-drug interactions [28, 31, 35].

Due to a rising number of new HIV diagnoses in older age education and guidance for geriatricians on when to screen patients for HIV is paramount [7, 29, 40]. Many older people will have grown up during the HIV epidemic and will recall the advertising campaigns many of which focussed on specific groups such as gay men or Black people from Africa indirectly contributing to the idea that White heterosexual people are at negligible risk of acquiring HIV, potentially explaining the rising rates within this group [6, 7, 40]. Advancements in HIV care has resulted in less public health campaigns resulting in low levels of knowledge about HIV amongst the general public but specifically amongst older heterosexual people [40, 41]. Stigma remains a large problem not only around HIV but also LGBTQ + relationships, sex outside of a long-term relationship or sex in later life resulting in healthcare professionals’ fear of offending older people by offering a test consequentially leading to a barrier to testing as well as older peoples’ potential embarrassment with them being less used to discussing sex and relationships with healthcare professionals [40, 42–44]. Meanwhile, whilst many younger patients may utilise sexual health services to undergo HIV testing the majority of testing in older people across Europe is performed within Primary Care [42–46]. This is important as many older people may not feel confident in attending sexual health services and therefore borrowing strategies from Primary Care such as offering routine HIV testing for all new patients or as part of a standard annual health check regardless of risk could be applied to all people attending geriatric medicine services helping to reduce stigma as well as identifying those undiagnosed particularly older women [29, 40, 42–44, 47].

When to refer an older person living with HIV to a geriatrician is also not well defined which is not unsurprising as the criteria for referring to a geriatrician generally varies not only across Europe but also within individual countries [48, 49]. Some centres may use chronological age whilst others favour alternate measures looking at indicators of biological age such as frailty scores [48, 49]. Interestingly whilst typical ageing research arbitrarily regards older people as being over 65 the literature on ageing HIV populations typically uses 50 based on the original age stratification of HIV set by the US Centers for Disease Control (CDC) [1, 50]. This age continues to be used today not only for this reason, but also as studies have demonstrated that people over 50 living with HIV are less likely to achieve a complete immunological response despite concordance to treatment when compared to younger people as well as 50 being shown to be the age when the main causes of mortality change to causes unrelated to HIV [1]. Given this is an evolving field this age cut-off should persist for now though referral processes should also follow EACS recommendations and calculate a frailty score, with the Fried frailty phenotype (FFP) being the most validated amongst people living with HIV [11, 15, 17, 51]. However, it must be noted that the FFP does not consider the broader elements of frailty such as psychosocial functioning or cognition and whilst other frailty scores do consider these they are not as well validated in people living HIV or in people under 65 [11, 51, 52]. Therefore, at present whilst further research is conducted referrals to geriatricians should be done based on local agreement with referral processes incorporating information on age, frailty score, presence of geriatric syndromes and any other concerns of the person living with HIV, their family and friends or the referrer themselves.

How best to deliver the care to older people living with HIV is also unclear as there is no agreed standardised model [17]. A survey of 27 HIV clinicians in the USA described them wanting assistance with managing multimorbidity (median: 85, range 65–100), polypharmacy (83, 51–100), cognitive impairment (80, 68.5–96), and mood disorders (80, 67–92) which are often common conditions identified in older people living with HIV and which geriatricians are well versed in [17, 18, 53]. They also reported a desire for the development of specific guidelines for older people living with HIV (68%) and access to more training on managing older people (60%) [53]. Similar findings have also been reported in Europe with HIV clinicians recognising the importance of geriatricians in managing frailty and multimorbidity [12, 54]. At present it is clear that no group of health professionals can manage the care of older people living with HIV independently so a collaborative service model remains the most appropriate due to the low levels of confidence, knowledge and experience amongst geriatricians and the lack of experience of HIV clinicians in managing frail older people. Several joint HIV and geriatric medicine clinics exist already internationally such as the ‘Sage Clinic’ at the Royal Free Hospital in London, UK, the ‘Silver Clinic’ in Brighton, UK and the ‘Golden Compass Programme’ in San Francisco, USA demonstrating positive outcomes from collaborative working [17, 18, 55]. However, this model may not be feasible everywhere due to issues of either supply or demand, with alternatives including incorporating geriatricians into multidisciplinary meetings about complex patients or having clear referral pathways into existing services for HIV clinicians to follow.

Increasing education on how to manage people living with HIV is fundamental to try and ensure geriatricians are comfortable due to the low levels reported with the creation of learning tools highlighted as important due to many currently lacking exposure in their current practice [25–30]. Another option would be for interested geriatric medicine trainees to spend time working in HIV medicine to gain experience, similar to an Orthogeriatrics or Oncogeriatrics model, with the elective depicted in this review being positively received by both the trainee and the supervising HIV clinicians [25, 56]. Education is important to reduce stigma, improve healthcare professionals’ attitudes towards people living with HIV and to provide better care, for example increasing awareness of drug-drug interactions [3, 6, 17, 21, 28, 57]. Basic education about HIV with specific focus on older people should be incorporated into geriatric medicine curricula to ensure all geriatricians gain some exposure [58]. Finally, it may be beneficial for HIV organisations such as EACS to come together with geriatric medicine bodies like the European Geriatric Medicine Society (EuGMS) to author joint standards and guidelines, promoting interspeciality working and holistic care [15, 16].

Finally, this review has also highlighted that geriatricians require more cultural competency training on managing LGBTQ + people regardless of HIV status [26]. Currently, gay and bisexual men make up a large number of older men living with HIV with sex between men still contributing to a significant proportion of new cases [32]. A 2010 study found that more than half of lesbian, gay, and bisexual respondents and 70% of transgender respondents had experienced discrimination by healthcare providers ranging from biases, incorrect assumptions, derogatory statements to refusal of care [59]. LGBTQ + older adults may delay or avoid health care because of fear of or previous experiences of discrimination while others may hide their identity when using healthcare services [60]. Given that the number of older LGBTQ + people in Europe is expected to double by 2030 geriatricians not only have a responsibility to educate themselves about the health conditions common amongst older LGBTQ + people, but also their lived experiences and be actively involved in ensuring accessible culturally sensitive services through public policy and societal guidelines with specific focus made to ensure trans people are well represented [26, 60–63].

As a scoping review the absence of methodological quality appraisal limits its ability to provide validated recommendations, however given the aim was to consolidate the views of geriatricians the impact of this should be minimal. All studies were conducted in the USA where cultural, political and healthcare model factors may impact the views of respondents and limit its generalisability [64–66]. However, given the knowledge gap it still has the potential to inform providers in Europe. There were only six publications, providing data from four separate studies with only one of the four having the primary aim being to ascertain the views of geriatricians on caring for people living with HIV [27, 28, 30]. Another limitation is that five of the six publications consist of conference abstracts [25–27, 29, 30]. Whilst conference abstracts may not contain detailed information, and determining the dependability of the results is challenging their inclusion was important due to the paucity of data available resulting in an increased in comprehensiveness and decreasing the impact of publication bias [67]. Half of the included publications were published within the last 5 years but as mentioned previously despite one having rich data the remaining three publications all by Hughes have less relevance due to their age [25–30]. The review was strengthened by not limiting studies by language meaning the chances of omitting those from lower and lower-middle income countries was reduced [68]. Therefore, this review provides an encompassing review of the literature on the views and experiences of geriatricians on managing people living with HIV and clearly demonstrates a scarcity of high-level evidence providing grounds for future high-quality research.

There is a lack of knowledge on this topic outside of the USA and more international studies are required due to differing healthcare systems and HIV prevalence between countries [64–66]. Health care systems will impact both how people living with HIV experience care as well as how geriatricians experience working within it [64–66]. Geriatric medicine training also varies between countries with some having more formal structured training programmes than others prompting the recent creation of a standardised European postgraduate curriculum [48, 58, 69]. Exploring the views of geriatricians across Europe is crucial to see whether the experiences reported are generalisable and not specific to the USA. This does not however consider the situation in lower and lower-middle income countries with a high prevalence of HIV such as in Sub-Saharan Africa where despite the rising numbers of older people living with HIV there remains very few geriatricians which must also be explored [66, 70].

This article provides a comprehensive review of the existing literature regarding the views of geriatricians on managing older people living with HIV providing the groundwork for future high-quality research and intervention strategies on how best to support people living with HIV as they age. Future steps comprise gaining an international view, developing educational tools for geriatricians including creating clinical practice guidelines. These are required before we can support recommendations for geriatricians play a dominant role in the care of older people living with HIV and will inform decisions on how best to structure HIV services across Europe to manage this ageing cohort in the near future.

## Supplementary Information

Below is the link to the electronic supplementary material.

Supplementary file1 (DOCX 26 KB)

Supplementary file2 (PDF 120 KB)

## Data Availability

All data relevant to the study are included in the article or uploaded as online supplemental information.
