# Supplementary material for: How do geriatricians feel about managing older people living with HIV? A scoping review
Source: Eur Geriatr Med. 2022 Apr 9;13(4):987–97. doi: 10.1007/s41999-022-00642-4 (PMC9378329; doi:10.1007/s41999-022-00642-4)
Supplement: Supplementary file 2 — Supplementary file2 (PDF 120 KB) [file 41999_2022_642_MOESM2_ESM.pdf]

People First Charter: recommended terminology for research and publications related to HIV

People living with HIV are not just hosts for a virus, vectors of infection, or trial subjects - they have awareness and agency. People living with HIV have been prominent in pushing for better medical care, treatment expansion and have been collaborators in the medical progress made. The language used to refer to people with HIV in research and publications, should reflect this.

We recognise that language is not universal, and that there are always challenges when translating words, including medical terminology. However, we believe that there are some terms that should no longer be used and others that we should consider moving away from.

This document is intentionally brief and designed as a first stop for people and organisations seeking to use People First language. More information about appropriate language is available from UNAIDS [1], NHIVNA [2] and The Salamander Trust [3]. Additional resources will be added regularly.

Please avoid	Alternatives
AIDS patient	Person with complications of advanced HIV, person with an AIDS-defining illness
AIDS test	HIV test
AIDS virus	HIV virus
Catch HIV	Acquire HIV
Compliant	Taking medication as recommended, adherent, concordant
Contagious/infectious	Person with transmittable HIV or detectable viral load
Dirty or clean in the context of people or injecting equipment	Just don’t use! Shared needles, injecting equipment or drug paraphernalia acceptable
HIV-infected person, people, individual(s), populations	Person/people living with HIV<br>HIV-positive individual(s)
Prostitute, prostitution	Sex worker, transactional sex
**Consider avoiding**	**Alternatives**
Abbreviations	Avoid abbreviating e.g. people who inject drugs (PWID), women living with HIV (WLWH) if possible
Co-infected person or people	Person living with HIV and <additional condition> e.g. person living with HIV and hepatitis B. Treating ‘HIV/hepatitis co-infection’ or living with HIV/HBV is acceptable, treating the ‘HIV/hepatitis co-infected’ is not
Detectable or viraemic patients	People with a detectable HIV-RNA or viral load, or people with viraemia
Ending HIV, ending AIDS	Ending HIV transmission, ending late HIV presentation or preventable HIV-related deaths
HIV exposed infant<br>HIV exposed uninfected infant	Infant exposed to HIV<br>HIV-negative infant exposed to HIV
Intravenous drug user/IVDU; drug addict; drug abuser	People who inject drugs; People who use drugs
Spread, infect	Transmit, pass on
HIV deaths	HIV-related mortality or HIV-related
Mother to child transmission	Vertical transmission, perinatally acquired HIV
Number of infections	Number of HIV diagnoses, number diagnosed with HIV, number of transmissions
People failing therapy; failing patients	People experiencing treatment failure, patients for whom treatment is ineffective
Risk group or transmission risk	Mode of HIV acquisition or acquisition risk’

Author: Laura Waters
Reviewers: Yvonne Gilleece, Caroline Sabin, Jo Josh & Matthew Hodson

Poorly adherent	Experiencing or reporting difficulty in taking medication regularly; experiencing challenges with adherence
Resistant patients	People with resistant virus
Serodiscordant	Serodifferent, partners with differing HIV status
Trial subjects	Trial participants, volunteers
Unprotected sex	Sex without a condom/condomless sex, sex without PrEP use
Zero infections	Zero transmissions, zero new cases of HIV/newly acquired HIV

There is ongoing debate about the appropriateness of 'patients' as opposed to 'clients' or 'service users'. We advise that, currently, all are acceptable but, ideally, people accessing services should be asked about their preferred terminology. UK research in mental health services show that people prefer 'patient' when consulted by a psychiatrist or nurse, and 'patient' or 'client' when consulted by social workers and occupational therapists; 'service' user was not preferred [4].

References

1) UNAIDS Terminology Guidance 2015; accessed at https://www.unaids.org/sites/default/files/media_asset/2015_terminology_guidelines_en.pdf 18th October 2021
2) Watson S et al. The language of HIV: a guide for nurses. HIV Nursing 2019; 19(2): BP1–BP4.
3) Salamander Trust. The Power of Language. Accessed at https://salamandertrust.net/project/the-power-of-language/ 18th October 2021
4) Simmons P et al. Service user, patient, client, user or survivor: Describing recipients of mental health services. The Psychiatrist 2010; 34(1): 20-23.

Author: Laura Waters
Reviewers: Yvonne Gilleece, Caroline Sabin, Jo Josh & Matthew Hodson
